# Impact of the Nature and Size of the Polymeric Backbone on the Ability of Heterobifunctional Ligands to Mediate Shiga Toxin and Serum Amyloid P Component Ternary Complex Formation

**DOI:** 10.3390/toxins3091065

**Published:** 2011-08-25

**Authors:** Pavel I. Kitov, Eugenia Paszkiewicz, Joanna M. Sadowska, Zhicheng Deng, Marya Ahmed, Ravin Narain, Thomas P. Griener, George L. Mulvey, Glen D. Armstrong, David R. Bundle

**Affiliations:** 1 Department of Chemistry, Alberta Ingenuity Centre for Carbohydrate Science, University of Alberta, Edmonton AB, Canada T6G 2G2; Email: eugeniap@ualberta.ca (E.P.); joanna.sadowska@ualberta.ca (J.M.S.); 2 Department of Chemical Engineering, University of Alberta, Edmonton AB, Canada; Email: zdeng@ualberta.ca (Z.D.); marya1@ualberta.ca (M.A.); narain@ualberta.ca (R.A.); 3 Department of Microbiology, Immunology, and Infectious Diseases, Alberta Ingenuity Centre for Carbohydrate Science, University of Calgary, Calgary, AB, Canada T2N 4N1; Email: gmulvey@ucalgary.ca (G.L.M.); armstrong@ucalgary.ca (G.D.A.)

**Keywords:** *E. coli* O157:H7, multivalent inhibitors, P^k^-trisaccharide, Gb_3_

## Abstract

Inhibition of AB_5_-type bacterial toxins can be achieved by heterobifunctional ligands (BAITs) that mediate assembly of supramolecular complexes involving the toxin’s pentameric cell membrane-binding subunit and an endogenous protein, serum amyloid P component, of the innate immune system. Effective *in vivo* protection from *Shiga* toxin Type 1 (Stx1) is achieved by polymer-bound, heterobifunctional inhibitors-adaptors (PolyBAITs), which exhibit prolonged half-life in circulation and by mediating formation of face-to-face SAP-AB_5_ complexes, block receptor recognition sites and redirect toxins to the spleen and liver for degradation. Direct correlation between solid-phase activity and protective dose of PolyBAITs both in the cytotoxicity assay and *in vivo* indicate that the mechanism of protection from intoxication is inhibition of toxin binding to the host cell membrane. The polymeric scaffold influences the activity not only by clustering active binding fragments but also by sterically interfering with the supramolecular complex assembly. Thus, inhibitors based on *N*-(2-hydroxypropyl) methacrylamide (HPMA) show significantly lower activity than polyacrylamide-based analogs. The detrimental steric effect can partially be alleviated by extending the length of the spacer, which separates pendant ligand from the backbone, as well as extending the spacer, which spans the distance between binding moieties within each heterobifunctional ligand. Herein we report that polymer size and payload of the active ligand had moderate effects on the inhibitor’s activity.

## 1. Introduction

Enteric infections with Shigatoxigenic *Escherichia coli* (STEC), particularly the O157:H7 strain, is the leading cause of hemolytic-uremic syndrome (HUS) in industrialized countries [1]. HUS is a term for an acute form of renal disease that commonly manifests itself as hemolytic anemia, acute renal failure, thrombocytopenia, and central nervous system impairment. Most symptoms of HUS are mediated by exotoxins called *Shiga* toxins (Stx) that enter the circulation through an eroded intestinal epithelium and are rapidly absorbed in target tissues such as the kidney and the central nervous system, as well as inflicting serious systemic damage [2]. 

*Shiga* toxins are a group of closely related bacterial toxins that are serologically differentiated into two types, Stx1 and Stx2 with ~60% homology, and a number of variants differing by just a few amino acids. Stx are potent cytotoxins with ribosomal deadenylase activity that cause cell death through activating pro-apoptotic signals by inducing an endoplasmic reticulum stress response in susceptible tissues. The Stx host cell receptor is the P^k^ trisaccharide head group [Gal(α1-4)Gal(β1-4)Glc(β1-*O*)] of the glycolipid, Gb_3_. 

Since outbreaks of STEC infections can be contained by sanitary measures and by monitoring the water and food supply the incidence of HUS is relatively low, hence, immunization of the general population cannot be justified given the safety constraints of a vaccine. Treatment of infected patients is needed to prevent progression of the infection to HUS. However, despite tremendous advances in understanding STEC pathogenesis the clinical options for treatment remain limited to mainly supportive strategies. Antibiotics can stimulate further Stx production and, therefore, are presently not recommended [3,4]. Most notable, an earlier attempt to develop a Stx-targeting therapy for STEC-induced HUS was an orally administered indigestible affinity agent, Synsorb-P^k^, designed to sequester Stx in the intestine before Stx entered the circulation. Unfortunately, this approach failed to demonstrate significant efficacy in a Phase 2 clinical trial [5]. Recently, passive immunization with monoclonal antibodies raised against Stx1 [6] and Stx2 [7] has been shown to be effective in animal models and a humanized monoclonal antibody urtoxazumab against Shiga-like toxin 2 is undergoing clinical trials [8]. Compounds that affect intracellular trafficking of Stx have shown efficacy *in vitro* and *in vivo* [9,10]. This suggests that a soluble injectable antitoxin agent might be able to prevent or lessen the severity of HUS. 

**Figure 1:**
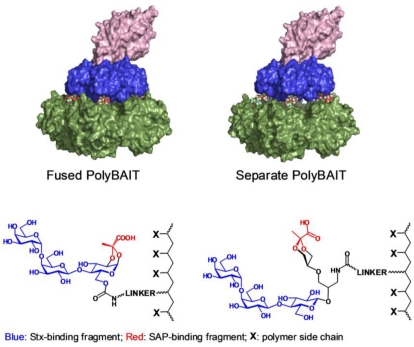
Molecular model of supramolecular complexes between Stx1 and SAP mediated by PolyBAIT. SAP: Green surface; Stx1-B_5_ subunit: Blue surface; Stx1-A subunit: Pink surface. Left panel: PolyBAIT with fused binding fragments; Right panel: PolyBAIT with separate binding fragments. Polymer atoms omitted for clarity. Molecular representation was rendered with PyMol (www.pymol.org).

Recently, we demonstrated the *in vivo* efficacy of a polyacrylamide-based pre-ordered heterobifunctional ligand named PolyBAIT that induces the formation of face-to-face complexes between the *Shiga* toxin pentameric B-subunit (Stx1-B_5_) and an endogenous human serum protein, serum amyloid P component (SAP, Figure 1), thereby protecting mice from intoxication by Stx1 [11]. SAP is a serum circulating pentraxin, a pentameric doughnut-shaped protein, that topologically matches the pentameric carbohydrate binding subunit of Stx1. The ligand-mediated ternary complex formation with SAP inhibits the cell-recognition domain of Stx1 and facilitates safe disposal of the complex in the liver [11].

Herein we report the synthesis and activity evaluation of a series of glycoconjugates containing a ligand with dual specificity for Stx1 and SAP linked to the polymeric scaffolds, polyacrylamide and *N*-(2-hydroxypropyl) methacrylamide (HPMA). We employ narrow molecular weight range polymeric scaffolds obtained by RAFT polymerization in order to investigate the impact of the nature and the size of the polymeric backbone and the linker on the activities of the polymeric heterobifunctional ligands. 

## 2. Materials and Methods

### 2.1. Synthesis of Heterobifunctional Inhibitors

Commercially available reagents were used as supplied without further purification. Evaporation and concentration *in vacuo* was conducted under water-aspirator pressure. All solution-phase reactions were carried out under nitrogen atmosphere. Reactions were monitored by analytical thin-layer chromatography (TLC) with pre-coated silica gel 60 F_254_ glass plate (Merck). Plates were visualized under UV light or stained by treatment with either cerium ammonium molybdate solution or 5% sulfuric acid in ethanol followed by heating at 180 °C. Purification of products was conducted by column chromatography using *silica gel* SiliaFlashF60 (40–63 μm, 60 Å) from *SiliCycle^®^* Inc. IR data were recorded on a *Nic–Plan IR Microscope* (solid film); only signals corresponding to functional groups indicative to the structure are reported. NMR spectra were recorded at 500 or 600 MHz, at 27 °C in CDCl_3_ or D_2_O. Chemical shifts are referenced to residual solvent (CDCl_3_) at 7.24 p.p.m. for ^1^H and 77.0 p.p.m. for ^13^C and relative to 0.1% external acetone at 2.225 p.p.m. for ^1^H for solutions in D_2_O. Electrospray ionization mass spectra were recorded on a Micromass Zabspec TOF-mass spectrometer. 

**Prop-2-ynyl 2-(2-(2-*t*-butyloxycarbonylaminoethoxy)ethoxy)ethylcarbamate (1).** To a solution of *tert*-butyl 2-[2-(2-aminoethoxy)ethoxy]ethylcarbamate [12] (698 mg, 2.81 mmol) in dry DCM (3 mL) at −78 °C triethylamine (0.85 g, 8.4 mmol) was added followed by propargyl chloroformate (522 mg, 4.4 mmol). The mixture was allowed to warm to room temperature and then stirred for 1.5 h. The mixture was diluted with DCM, washed with brine, concentrated and chromatographed on silica gel using hexane-acetone (7:3) to yield product **1** as a clear syrup (689 mg, 74%); ^1^H-NMR (CDCl_3_) δ: 5.63 (bs, 1 H, NH), 5.26 (bs, 1 H, NH), 4.93 (d, 2 H, *J* 2.3 Hz, CH_2_), 3.88–3.86 (m, 4 H, OCH_2_), 3.83–3.76 (m, 4 H, OCH_2_), 3.66–3.60 (m, 2 H, NCH_2_), 3.57–3.52 (m, 2 H, NCH_2_), 2.70 (t, 1 H, CH), 1.70 (s, 9 H *t*Bu); ESI HRMS: *m*/*z*: calcd for C_15_H_26_N_2_O_6_Na: 353.1683; found: 353.1681 [M + Na]^+^.

**3-*O*-Acetyl-6-*O*-(10-oxo-3,6,11-trioxa-9-azatetradec-13-ynyl)carbamoyl-4-*O*-(2,3,4,6-tetra-*O*-acetyl-β-D-galactopyranosyl)-1,2-*O*-[(*S*)-1-(methoxycarbonyl)ethylidene]-α-D-glucopyranoside (3).** Alkyne **1** (545 mg, 1.65 mmol) was dissolved in TFA (1.6 mL) and left at room temperature for 1 h. The mixture was concentrated, treated with Et_3_N and concentrated again then 3-*O*-acetyl-6-*O*-(4-nitrophenyloxycarbonyl)-4-*O*-(2,3,4,6-tetra-*O*-acetyl-β-D-galactopyranosyl)-1,2-*O*-[(*S*)-1-(methoxycarbonyl) ethylidene]-α-D-glucopyranoside **2** [11] (886 mg, 1.105 mmol) was added. The mixture was dissolved in dry DCM (10 mL) and triethylamine (310 μL, 2.21 mmol) was added. The reaction mixture was stirred at room temperature for 2 h. The mixture was concentrated and chromatographed on silica gel using hexane-acetone (2:1 to 1:1) to provide the title compound **3** as a white foam (0.93 g, 94%); ^1^H-NMR (CDCl_3_) δ: 5.76 (d, 1 H, *J*_1,2_ 5.13 Hz, H-1) 1.53–1.52 (m, 1 H, H-3), 5.46–5.40 (m, 1 H, NH), 5.38 (dd, 1 H, H *J*_3',4'_ 3.5 Hz, *J*_4',5'_ 0.7 Hz, H-4'), 5.18 (dd, 1 H, *J*_1',2'_ 7.0 Hz, *J*_2',3'_ 10.4 Hz, H-2'), 5.01 (dd, 1 H, H-3'), 4.68 (d, 2 H, *J* 2.2 Hz, CH_2_), 4.62 (d, 1 H, H-1'),4.36–4.33 (m, 1 H, H-2), 4.24 (dd, 1 H, *J*_5,6a_ 2.2 Hz, *J*_6a,6b_ 11.7 Hz, H-6a), 4.18–4.10 (m, 3 H, H-6b, H-6'a, H-6'b), 3.96–3.93 (m, 1 H, H-5'), 3.92–3.88 (m, 1 H, H-5), 3.76 (s, 3 H, OCH_3_), 3.64–3.54 (m, 8 H, OCH_2_), 3.42–3.36 (m, 4 H, NCH_2_), 2.48 (t, 1 H, CH), 2.18, 2.10, 2.03 1.98 (5 s, 15 H, 5 OAc), 1.76 (s, 3 H, CH_3_); ESI HRMS: *m/z*: calcd for C_37_H_52_N_2_O_23_Na: 915.2853; found 915.2846 [M + Na]^+^.

**1,2-*O*-[(*S*)-1-(carboxy)ethylidene]-4-*O*-(β-D-galactopyranosyl)-6-*O*-(10-oxo-3,6,11-trioxa-9-azatetradec-13-ynyl)carbamoyl-α-D-glucopyranoside (4).** Disaccharide **3** (480 mg, 0.537 mmol) was dissolved in dry methanol (4 mL) and 1 M NaOMe (0.54 mL) was added. The solution was stirred at room temperature for 16 h. The mixture was concentrated and dissolved in water. After 0.5 h TLC indicated that hydrolysis of the methyl ester was complete. The mixture was neutralized with 5 M acidic acid and lyophilized to provide the product **4** as white foam (387 mg, 94%). ^1^H-NMR (D_2_O) δ: 5.62 (d, 1 H, *J*_1,2_ 5.0 Hz, H-1), 4.67 (d, 1 H, *J* 2.1 Hz, CH_2_), 4.44 (d, 1 H, *J*_1',2'_ 7.8 Hz, H-1'), 4.41–4.37 (m, 2 H, *J*_2,3 _4.1 Hz, *J*_6a,6b_ 11.9 Hz, H-3, H-6a), 4.24 (dd, 1 H, *J*_5,6b_ 5.2 Hz, H-6b), 4.18 (dd, 1 H, H-2), 4.06–4.01 (m, 1 H, H-5), 3.91 (d, 1 H, *J*_3',4'_ 3.4 Hz, H-4'), 3.82–3.60 (m, 13 H, H-4, H-3', H-5', H-6'a, H-6'b, 4 × OCH_2_), 3.55 (dd, 1 H, H-2'), 3.36–3.32 (m, 4 H, 2 × NCH_2_), 2.9 (t, 1 H, CH), 1.64 (s, 3 H, CH_3_). ESI HRMS: *m/z*: calcd for C_26_H_39_N_2_O_18_: 667.2203; found 667.2205 [M *−* H]^−^.

**1,2-*O*-[(*S*)-1-(carboxy)ethylidene]-4-*O*-[4-*O*-(α-D-galactopyranosyl)-β-D-galactopyranosyl]-6-*O*-(10-oxo-3,6,11-trioxa-9-azatetradec-13-ynyl)carbamoyl-α-D-glucopyranoside (5).** Compound **4** (379 mg, 0.537 mmol) was dissolved in water (5.34 mL) and the pH was adjusted to 7.5 by addition of solid NaHCO_3_. Then 1 M HEPES buffer (pH: 8, 3.2 mL) was added followed by 0.4 M DTT solution (500 μL) and alkaline phosphatase (1 U/μL, 50 μL, Sigma). UDP-glucose (578 mg, 0.849 mmol) was dissolved in the reaction mixture and the fusion enzyme GalT/UDP-4'-epimerase [21] (950 μL) was added. The reaction mixture was incubated at 37 °C. After 24 h the reaction mixture was treated with Dowex H^+^ resin, filtered and freeze-dried. NMR of the crude product confirmed the reaction was complete. The mixture was purified on silica gel using DCM-methanol (2:1 with 2% acetic acid) to afford after 2 passages pure trisaccharide **5** (270 mg, 61%). Impure fractions were further purified on HPLC (C_18_) using water (with 0.1% TFA)–acetonitrile to yield an additional amount of trisaccharide (51 mg, 11%); ^1^H-NMR (D_2_O) δ: 5.64 (d, 1 H, *J*_1,2_ 5.0 Hz, H-1), 4.94 (d, 1 H, *J*_1'',2''_ 4.0 Hz, H-1''), 4.67 (d, 2 H, *J* 2.2 Hz, CH_2_), 4.51 (d, 1 H, *J*_1',2'_ 7.8 Hz, H-1'), 4.41–4.33 (m, 3 H, H-3, H-6a, H-5''), 4.28–4.21 (m, 2 H, H-2, H-6b), 4.04–4.00 (m, 3 H, H-5, H-4', H-4''), 3.94–3.90 (m, 2 H, H-6'a, H-3''), 3.85–3.75 (m, 4 H, H-4, H-5', H-6'b, H-2''), 3.72–3.66 (m, 7 H, H-3', H-6''a, H-6''b, 2 × OCH_2_), 3.63–3.56 (m, 5 H, H-2', 2 × OCH_2 _), 3.34–3.32 (m, 4 H, 2 × NCH_2_), 2.90 (t, 1 H, CH), 1.69 (s, 3 H, CH_3_). ESI HRMS: *m/z*: calcd for C_32_H_50_N_2_O_23_: 829.2732; found: 829.2734 [M − H]^−^.

**(*R*,*S*)-5-(3-azido-2-hydroxypropoxy)-*N*,*N*,2-trimethyl-1,3-dioxane-2-carboxamide (7).** Epoxide **6** [13] (4.595 g, 18.734 mmol) was dissolved in dry DMF (20 mL) then sodium azide (3.66 g, 56.2 mmol) and ammonium chloride (3.01 g, 56.2 mmol) were added and the mixture was stirred for 5 h at 50 C then overnight at room temperature. Water (100 mL) was added and the mixture was extracted with ethyl acetate (3 × 50 mL). Combined organic layers were washed with saturated NH_4_Cl and brine, then dried with MgSO_4_, filtered and concentrated. The residue was purified on silica gel using hexane-acetone (3:1) to provide the title compound **7** as a clear syrup (2.9 g, 54%). ^1^H-NMR (CDCl_3_) δ: 4.10–4.06 (m, 2 H, H-4_e_, H-6_e_), 3.92–3.82 (m, 1 H, OCH), 3.70–3.48 (m, 5 H, H-4_a_, H-5, H-6_a_, OCH_2_), 3.40–3.28 (m, 2 H, NCH_2_), 3.24 and 3.02 (2s, 6 H, NMe_2_), 1.52 (s, 3 H, CH_3_); ESI HRMS: *m/z*: calcd for C_11_H_20_N_4_O_5_Na: 311.1326; found: 311.1329 [M + Na]^+^.

**(*R*,*S*)-5-{3-azido-2-[2,3,6-tri-*O*-acetyl-4-(2,3,4,6-tetra-*O*-acetyl-β-D-galactopyranosyl)-β-D-glucopyranosyloxy]propoxy}-*N*,*N*,2-trimethyl-1,3-dioxane-2-carboxamide (9).** Azide **7** (1.05 g, 3.64 mmol) and lactosyl trichloroacetimidate **8** [14] (3.37 g, 4.32 mmol) were dried together under vacuum overnight then dissolved in dry DCM (30 mL) and powdered molecular sieves 4 Å were added and the mixture was stirred under argon for 0.5 h and cooled to 0 °C. TMSOTf (50 μL, 61 mg, 0.27 mmol) was added slowly then the ice bath was removed and stirring continued at room temperature. After 40 min the mixture was neutralized with a drop of Et_3_N, filtered through celite, concentrated and chromatographed on silica gel using hexane-acetone (3:1 to 3:2) to provide the title product **9** as white foam (2.4 g, 69 %); ^1^H-NMR (CDCl_3_) δ: 5.36–5.34 (m, 1 H, *J*_3',4'_ 3.4 Hz, H-4'), 5.20−5.16 (m, 1 H, H-3), 5.13–5.08 (m, 1 H, *J*_1',2'_ 7.8 Hz, H-2'), 4.98–4.94 (m, 1 H, H-3'), 4.90-4.84 (m, 1 H, *J*_1,2_ 7.9 Hz, H-2), 4.63 (d, 1 H, H-1), 4.60-4.56 (m, 1 H, H-6a), 4.52–4.49 (m, 1 H, H-1'), 4.15–4.02 (m, 5 H, H-4e_pyr_, H-6e_pyr_, H-6b, H-6a', H-6b'), 3.90–3.85 (m, 1 H, H-5'), 3.83–3.68 (m, 2 H, H-4, CH), 3.65–3.44 (m, 6 H, H-5, H-4a_pyr_, H-5_pyr_, H-6a_pyr_, OCH_2_), 3.41–3.35 and 3.30–3.22 (2 m, 2 H, NCH_2_), 3.22 and 3.02 (2 s, 6 H, NMe_2_), 2.16–1.97 (ms, 21 H, 7 × OAc), 1.50 (s, 3 H, CH_3_). ESI HRMS: *m/z*: calcd for C_47_H_54_N_4_O_22_Na: 929.3; found: 929.3 [M + Na]^+^.

**(*R*,*S*)-5-{3-acetamido-2-[2,3,6-tri-*O*-acetyl-4-(2,3,4,6-tetra-*O*-acetyl-β-D-galactopyranosyl)-β-D-glucopyranosyloxy]propoxy}-*N*,*N*,2-trimethyl-1,3-dioxane-2-carboxamide (10).** To a solution of azide **9** (945 mg, 1.04 mmol) in dry THF (19 mL) triphenylphosphine (423 mg, 1.6 mmol) was added and the mixture was stirred at 65 °C for 6 h then water (2.5 mL) was added and the mixture was stirred at 65 °C for 6.5 h, then concentrated, co-evaporated with toluene and dried under vacuum. The residue was dissolved in pyridine (20 mL) and acetic anhydride (10 mL) was added then the mixture was left at room temperature overnight. The mixture was concentrated, co-evaporated with toluene and the residue was chromatographed on silica gel using hexane-acetone (1:1) to provide *N*-acetate **10** as a white foam (635 mg; 65 %); ^1^H-NMR (CDCl_3_) δ: 6.07 (t, 0.45 H, *J* 5.5 Hz, NH), 5.89 (t, 0.55 H, *J* 5.5 Hz, NH), 5.35 (dd, 1 H, *J*_3',4'_ 3.4 Hz, *J*_4',5'_ 0.3 Hz, H-4'), 5.18 (dd, 1 H, *J*_2,3_ = *J*_3.4 _= 9.4 Hz, H-3), 5.13–5.10 (m, 1 H, *J*_1',2'_ 7.9 Hz, *J*_2',3'_ 10.4 Hz, H-2'), 4.98 (dd, 1 H, H-3'), 4.88–4.83 (m, 1 H, H-2), 4.63 (d, 0.55 H, H-1), 4.60–4.56 (m, 1 H, H-6a), 4.55 (d, 0.45 H, H-1), 4.50 (d, 1 H, H-1'), 4.16–4.02 (m, 5 H, H-6b, H-6'a, H-6'b, H-4e_pyr_, H-6e_pyr_), 3.90–3.86 (m, 1 H, H-5'), 3.79–3.70 (m, 2 H, H-4, OCH), 3.66–3.41 (m, 7 H, H-5, H-4a_pyr_, H-5_pyr_, H-6a_pyr_, NCH, OCH_2_), 3.22 (s, 3 H, NCH_3_), 3.21–3.12 (m, 1 H, NCH), 2.17–2.04 (ms, 18 H, 6 x OAc), 1.96 (s, 6 H, NAc, OAc), 1.50 (2 s, 3 H, CH_3_); ESI HRMS: *m/z*: calcd for C_39_H_58_N_2_O_23_Na: 945.3323, found: 945.3315 [M + Na]^+^. 

**(*R*,*S*)-5-{3-(prop-2-yn)oxycarbonylamino-2-[4-(β-D-galactopyranosyl)-β-D-glucopyranosyloxy]propoxy}-2-methyl-1,3-dioxane-2-carboxylic acid (12).** Acetate **10** (186 mg, 0.2 mmol) was dissolved in methanol (4 mL) and transferred to a plastic Falcon tube, then 4 M NaOH (0.5 mL, 2 mmol) was added. The mixture was stirred at 80 °C. After 3 days the reaction was not complete as checked by NMR. More 4 M NaOH (0.1 mL, 0.4 mmol) was added and stirring was continued overnight at 85 °C. The mixture containing hydrolysis product **11** was treated with dry ice until the solution reached pH 8–9. Then propargyl chloroformate (40 μL, 0.4 mmol) was added and the mixture was stirred at room temperature. After 2 h the mixture was purified on HPLC (C-18) using water containing 0.1% TFA—acetonitrile gradient. Two fractions were isolated, concentrated and freeze-dried. First fraction was the product of incomplete hydrolysis of **10** (*R*,*S*)-5-{3-acetamido-2-[4-(β-D-galactopyranosyl)-β-D-glucopyranosyloxy]propoxy}-2-methyl-1,3-dioxane-2-carboxylic acid (11a) (54 mg, 45%), ^1^H-NMR (D_2_O) δ: 4.56 (d, 1 H, *J*_1,2_ 7.9 Hz, H-1), 4.44 (d, 1 H, *J*_1',2'_ 7.8 Hz, H-1'), 4.16 (dd, *J*_vic_ 4.5 Hz, *J*_gem _11.4 Hz, H-4e_pyr_, H-6e_pyr_), 4.02–3.99 (m, 1 H, CH), 3.95 (dd, 1 H, *J*_5,6a_ 1.7 Hz, *J*_6a,6b_ 12.1 Hz, H-6a), 3.06 (d, 1 H, J_3',4'_ 3.3 Hz, H-4'), 3.82 (m, 14 H, H-3, H-4, H-5, H-6b, H-2', H-3', H-5', H-6'a, H-6'b, H-4a_pyr_, H-5_pyr_, H-6a_pyr_, OCH_2_), 3.40–3.29 (m, 3 H, H-2, NCH_2_), 2.00 (s, 3 H, OAc), 1.00 (s, 3 H, CH_3_); ESI HRMS: *m/z*: calcd for C_23_H_39_NO_17_: 624.211; found: 624.2104 [M+ Na]^+^. This intermediate can be recycled.

The second fraction was the target compound **12** (59 mg, 46%); ^1^H-NMR (D_2_O) δ: 4.65 (bs, 2 H, CH_2 propargyl_), 4.56–4.50 (m, 1 H, *J*_1.2 _7.9 Hz, H-1), 4.42 (d, 1 H, *J*_1',2'_ 7.8 Hz, H-1'), 4.24–4.16 (m, 2 H, H-4e_pyr_, H-6e_pyr_), 4.00–3.88 (m, 3 H, *J*_3',4'_ 3.3 Hz, H-6a, H-4', CH), 3.86–3.50 (m, 14 H, *J*_2',3_ 9.9 Hz, H-3, H-4, H-5, H-6b, H-2', H-3', H-5', H-6'a, H-6'b, H-4a_pyr_, H-5_pyr_, H-6a_pyr_, OCH_2_), 3.30–3.24 (m, 3 H, H-2, NCH_2_), 2.90 (bs, 1 H, CH_propargyl_), 1.52 (s, 3 H, CH_3_); ESI HRMS: *m/z*: calcd for C_25_H_38_NO_18_: 640.2094; found: 640.2091 [M − H]^−^.

**(*R*,*S*)-5-{3-(prop-2-yn)oxycarbonylamino-2-[α-D-galactopyranosyl-(1-4)-β-D-galactopyranosyl-(1-4)-β-D-glucopyranosyloxy]propoxy}-2-methyl-1,3-dioxane-2-carboxylic acid (13).** Lactose derivative **12** (110 mg, 0.17 mmol) was dissolved in water (1.5 mL) and neutralized with dry NaHCO_3_. Then other components were added in the following order: 1 M HEPES buffer (pH: 8; 0.93 mL), DTT solution (0.4 M, 145 μL), alkaline phosphatase (1 U/μL, 14.5 μL, Sigma), UDP-glucose (157 mg, 0.257 mmol) and crude fusion enzyme GalT/UDP-4'-epimerase [21] (0.3 mL). The mixture was incubated for 40 h at 37 °C. The mixture was treated with Dowex H^+^ resin, filtered and purified on HPLC (C-18) using water with 0.1% TFA and acetonitrile gradient. The fraction containing product was concentrated and lyophilized to afford the product **13** as a white foam (116 mg, 85 %); ^1^H-NMR (D_2_O) δ: 4.92 (d, 1 H, *J*_1'',2''_ 4.0 Hz, H-1''), 4.66 (d, 2 H, *J* 1.8 Hz, CH_2_ _propargyl_), 4.56–4.52 ( m, 1 H, *J*_1,2_ 7.6 Hz, H-1), 4.50 (d, 1 H, *J*_1',2'_ 7.8 Hz, H-1'), 4.34–4.31 (m, 1 H, *J*_5'',6''a_ = *J*_5'',6''b_= 6.4 Hz, H-5''), 4.22 (m, 2 H, H-4e_pyr_, H-6e_pyr_), 4.03–4.00 (m, 2 H, *J*_3',4'_ 3.2 Hz, *J*_3'',4''_ 3.3 Hz, H-4', H-4''), 3.99–3.94 (m, 2 H, H-5, CH), 3.93–3.54 (m, 18 H, H-3, H-4, H-6a, H-6b, H-2', H-3', H-5', H-6'a, H-6'b, H-2'',H-3'', H-6''a, H-6''b, H-4a_pyr_, H-5_pyr_, H-6a_pyr_, OCH_2_), 3.38–3.27 (m, 3 H, H-2, NCH_2_), 2.89 (bs, 1 H, CH_propargyl_), 1.52 (s, 3 H, CH_3_); ESI HRMS: *m/z*: calcd for C_31_H_49_NO_23_Na: 826.2588; found: 826.2588 [M + Na]^+^.

**4-Nitrophenyl 2-(2-(2-(prop-2-ynyloxy)ethoxy)ethoxy)ethyl carbonate (15).** To a solution of 2-(2-(2-(prop-2-ynyloxy)ethoxy)ethoxy)ethanol **14** [15] (1.953 g, 10.37 mmol) and 4-nitrophenyl chloroformate (2.51 g, 12.5 mmol) in dry DCM pyridine (2 mL) was slowly added at room temperature. After 30 min the reaction was quenched by addition of methanol, diluted with DCM and washed with brine. The organic layer was collected, concentrated and the residue was chromatographed on silica gel using hexane acetone (3:1) to provide **15** as a clear syrup (3.345 g; 91%); ^1^H-NMR (CDCl_3_) δ: 8.30–8.26 and 7.42–7.37 (2 m, 4 H, C_6_H_4_), 4.46–4.42 (m, 2 H, CH_2_OCO), 4.20 (d, 2 H, *J* 3.4 Hz, CH_2_ _propargyl_), 3.84–3.80 (m, 2H, OCH_2_), 3.73–3.67 (m, 8 H, 4 × CH_2_), 2.43 (t, 1 H, CH_propargyl_); ESI HRMS: *m/z*: calcd for C_16_H_19_NO_8_: 376.1003; found: 376.1004 [M+ Na]^+^.

**(*R*,*S*)-5-{2-[β-D-galactopyranosyl-(1-4)-β-D-glucopyranosyloxy]-5-oxo-6,9,12,15-tetraoxa-4-azaoctadec-17-ynyloxy}-2-methyl-1,3-dioxane-2-carboxylic acid (16).** To a solution of **10** (545 mg, 0.59 mmol) in methanol (12 mL) in a plastic tube NaOH (4 M, 2.25 mL, 9 mmol) was added and the mixture was stirred at 80 °C for 3 days. The mixture was diluted with water and treated with dry ice until the solution pH was ~11 at which point a solution of 4-nitrophenylcarbonate **15** (0.6 g, 1.7 mmol) in methanol (2 mL) was added. The mixture was stirred overnight at room temperature. The reaction was not complete. Et_3_N (410 μL, 3 mmol) and more **15** (0.3 g, 0.85 mmol) were added and the mixture was stirred overnight. The reaction was not complete. The mixture was concentrated and purified by HPLC (C-18) using water with 0.1% of TFA and acetonitrile gradient to provide compound **16** (285 mg; 62%) as a white foam; ^1^H-NMR (D_2_O) δ: 4.55–4.54 (m, 1 H, *J*_1,2_ 7.9 Hz, H-1), 4.43 (d, 1 H, *J*_1',2'_ 7.8 Hz, H-1'), 4.23 (d, 2 H, *J* 2.4 Hz, CH_2propargyl_), 4.22–4.15 (m, 4 H, H-4e_pyr_, H-6e_pyr_, CH_2_OCO), 3.98–3.92 (m, 2 H, H-6a, CH), 3.91 (d, 1 H, *J*_3',4'_ 3.4 Hz, H-4'), 3.81–3.50 (m, 24 H, *J*_2',3'_ 9.9 Hz, H-3, H-4, H-5, H-6b, H-2', H-3', H-5', H-6'a, H-6'b, H-4a_pyr_, H-5_pyr_, H-6a_pyr_, 6 × OCH_2_), 3.37–3.26 (m, 3 H, H-2', NCH_2_), 2.89 (t, 1 H, CH propargyl), 1.50 (s, 3 H ,CH_3_); ESI HRMS: *m/z*: calcd for C_31_H_51_NO_21_Na: 796.2846; found: 796.2841 [M+ Na]^+^.

**(*R*,*S*)-5-{2-[α-D-galactopyranosyl-(1-4)-β-D-galactopyranosyl-(1-4)-β-D-glucopyranosyloxy]-5-oxo-6,9,12,15-tetraoxa-4-azaoctadec-17-ynyloxy}-2-methyl-1,3-dioxane-2-carboxylic acid (17).** Lactose derivative **16** (273 mg, 0.35 mmol) was dissolved in water (3 mL) and neutralized with dry NaHCO_3_. Then other components were added in the following order: 1 M HEPES buffer (pH: 8; 1.86 mL), DTT solution (0.4 M, 300 μL), alkaline phosphatase (1 U/μL, 30 μL, Sigma), UDP-glucose (320 mg, 0.52 mmol) and crude fusion enzyme GalT/UDP-4'-epimerase [21] (0.5 mL). The mixture was incubated for 24 h at 37 °C then treated with Dowex H^+^ resin, filtered and purified on HPLC (C-18) using water with 0.1% TFA and acetonitrile gradient. The fraction containing product was concentrated and lyophilized to afford the product **17** as a white foam (272 mg; 82 %); ^1^H-NMR (D_2_O) δ: 4.94 (m, 1 H, *J*_1'',2''_ 4.0 Hz, H-1''), 4.67–4.65 (m, 1 H, *J*_1,2_ 7.8 Hz, H-1), 4.50 (d, 1 H, *J*_1',2'_ 7.7 Hz, H-1'), 4.36-4.32 (m, 1 H, *J*_ 5'',6''a _= *J*_5'',6''b_ = 6.4 Hz, H-5''), 4.24 (d, 2 H, *J* 2.4 Hz, CH_2_ propargyl), 4.22–4.16 (m, 4 H, H-4e_pyr_, H-6e_pyr_, CH_2_OCO), 4.04–4.01 (m, 2 H, H-4', H-4''), 4.00-–3.52 (m, 30 H, H-3, H-4, H-5, H-6a, H-6b, H-2', H-3', H-5', H-6'a, H-6'b, H-2'', H-3'', H-6''a, H-6''b, H-4a_pyr_, H-5_pyr_, H-6a_pyr_, OCH, 6 × OCH_2_), 3.38–3.27 (m, 3 H, H-2, NCH_2_), 2.58 (t, 1 H, CH_propargyl_), 1.50 (s, CH_3_); ESI HRMS: *m/z*: calcd for C_37_H_61_NO_26_Na: 958.3374; found 958.3366 [M + Na]^+^.

***N*-[3-(*tert*-Butyloxycarbonylamino)propyl]methacrylamide (19).** To a solution of 1,3-diaminopropane (1 mL) in DCM (4 mL) methacrylic acid NHS ester (0.36 g, 1.96 mmol) was added. The mixture was stirred at room temperature for 15 min. The mixture was concentrated and the residue dissolved in methanol. To the solution di(*tert*-butoxy)carbonyl oxide (2.5 g, 10 mmol) was added followed by triethylamine (3 mL, 25 mmol). After 1 h the mixture was concentrated and the residue was chromatographed on silica gel using hexane-ethyl acetate (3:2) to give the title product **19** (0.2 g, 42%); ^1^H-NMR (CDCl_3_) δ: 6.74 (broad s, 1 H, NH), 5.73 (s, 1 H, CH_2_), 5.31 (s, 1 H, CH_2_), 4.96 (broad s, 1 H, NH), 3.35 (dd, 2 H, *J* 6.2 Hz, NCH_2_), 3.18 (dd, 2 H, *J* 5.9 Hz, NCH_2_), 1.96 (s, 3 H, CH_3_), 1.67–1.58 (m, 2 H, CH_2_), 1.43 (s, 9 H, *t*Bu). ESI HRMS: *m/z*: calcd for C_12_H_22_N_2_O_3_Na ([M + Na]^+^): 265.15226; found: 265.15212.

***tert*-Butyl 2-(2-((4-nitrophenoxy)carbonyloxy)ethoxy)ethylcarbamate (21).** 2-(2-Aminoethoxy)-ethanol (2.17 g; 20.6 mmol) was dissolved in methanol (10 mL) and di(*tert*-butoxy)carbonyl oxide (6.75 g; 30.9 mmol) was added followed by triethylamine (3.13 g, 30.9 mmol). After 30 min the mixture was concentrated and dried overnight under a vacuum provided by an oil pump. The residue was dissolved in dry DCM (20 mL) and *p*-nitrophenyl chloroformate (5.18 g, 25.7 mmol) was added followed by pyridine (3.26 g, 41.2 mmol). After 30 min the reaction was quenched by addition of methanol, then the mixture was diluted with DCM, washed with brine, concentrated and the residue was chromatographed on silica gel using hexane-acetone (4:1) to provide the product as slightly yellow syrup (7.146 g, 94%); ^1^H-NMR (CDCl_3_) δ: 8.24 and 7.40 (2 d, 4 H, C_6_H_4_), 4.90 (broad s, 1 H, NH), 4.43 (ddd, 2 H, *J* 4.6 Hz, *J* 2.9 Hz, OCH_2_), 3.77 (ddd, 2 H, OCH_2_), 3.58 (t, 2 H, *J* 5.2 Hz, NCH_2 _), 3.38–3.00 (m, 2 H, NCH_2_), 1.45 (s, 9 H, *t*-Bu). ESI HRMS: *m*/*z*: calcd for C_16_H_22_N_2_O_8_Na ([M + Na]^+^): 393.12684; found 393.12652.

**Copolymer of *N*-(2-hydroxypropyl)methacrylamide with methacrylamide 19 (20).** A solution of *N*-(2-hydroxypropyl)methacrylamide (502.5 mg, 3.5 mmol) and **19** (42 mg, 0.174 mmol) in degassed water (7 mL) was ultrasonicated under vacuum. A solution of ammonium persulfate (7 mg in 70 μL of water) was added to the mixture followed by an aq. solution of cysteamine hydrochloride (98 μL; 1 mg/100 μL). The reaction mixture was vortexed and incubated at 50 °C overnight then concentrated and dissolved in treated with TFA (8 mL). After 2 h the mixture was concentrated, the residue was dissolved in water, dialyzed extensively against deionized water, then freeze-dried to provide a white powder of aminated HPMA polymer **20** (445 mg, 82%); ^1^H-NMR (D_2_O) δ: 4.00–3.90 (broad s, 1 H, CHOH), 3.30–3.00 (m, 2.2 H, CH_2_N, CH_2_NH_2_), 2.00–1.60 (m, 2 H, CH_2_), 1.60–0.80 (m, 6.3 H, CH_2_, CH_3_). Integration of ^1^H-NMR signals indicated ~5% incorporation of propylenediamine. 

### 2.2. Elongation of Side Chains. Preparation of ***22*** and ***23***

To a solution of the polymer **20** (70 mg) in dry DMF (1 mL) the linker **21** (46 mg, 0.123 mmol) was added followed by triethylamine (25 mg, 0.25 mmol). The mixture was stirred overnight then diluted with water, dialyzed against deionized water and freeze-dried. The dry product was dissolved in TFA (2 mL) and stirred at room temperature for 30 min. The mixture was diluted with water (8 mL) carefully neutralized with dry sodium carbonate (1.33 g) in small portions. Then the solution was dialyzed against deionized water and lyophilized to give polymer **22** as white powder (62 mg); ^1^H-NMR (D_2_O) δ: 4.5 (bs, 8.3 H, CH_2_OCO), 3.90 (bs, 100 H, CHOH_HPMA_), 3.80 (bs, 21.8 H, OCH_2_), 3.30–2.95 (m, 230 H, NCH_2_), 2.20–0.60 (m, 840 H, CH_2_, CH_3_). 

Polymer **22** (87 mg, 0.029 mmol) was treated with linker **21** again as described for the synthesis of **22** to yield polymer **23** as white foam (78 mg); ^1^H-NMR (D_2_O) δ: 4.60–4.40 (m, 18.5 H, CH_2_OCO), 3.90 (broad s, 100 H, CHOH), 3.35–3.20 (m, 32 H, OCH_2_), 3.65 (broad s, 11 H, OCH_2_), 3.40–3.00 (m, 240 H, NCH_2_), 2.10–0.60 (m, 844 H, CH_2_, CH_3_).

### 2.3. Conjugation of Lactose Derivative. Preparation of Polymers ***24***, ***25***, and ***26***

To a clear solution of polymer **20** (52.5 mg, 0.018 mmol) and activated carbonate **2**  [11] (3.7 mg, 0.046 mmol) in dry DMF (1 mL) triethylamine (9.5 mg, 0.093 mmol) was added and the mixture was stirred at room temperature overnight. The reaction mixture was diluted with water and dialyzed against deionized water, then freeze-dried to afford the protected intermediate as white powder (67.5 mg). The intermediate was dissolved in dry methanol (2.5 mL) and NaOMe (0.5 M, 0.23 mL) was added to the solution. The mixture was stirred at room temperature for 3 h and concentrated. The residue was dissolved in water (2 mL) and stirred for 1 h then dialyzed against deionized water and lyophilized to provide the product  **24** as a white powder (50 mg); ^1^H-NMR (D_2_O) δ: 5.64 (d, 1 H, *J*_1,2 _ 4.8 Hz, H-1), 4.46 (d, 1 H, *J*_1',2' _ 7.7 Hz, H-1'), 4.43–4.36 (m, 2 H, H-3, H-6a), 4.30–4.15 (m, 2 H, H-2, H-6b), 4.08–4.00 (m, 1 H, H-5), 3.94–3.88 (m, 21 H, *J*_3',4'_ 2.9 Hz, H-4', CHOH), 3.85–3.68 (m, 4 H, H-4, H-5', H-6a', H-6b'), 3.64 (dd, 1 H, *J*_2',3'_ 9.9 Hz, H-3'), 3.56 (dd, 1 H, H-2'), 3.30–3.00 (m, 41 H, NCH_2_), 2.20–0.60 (m, 160 H, CH_2_, CH_3_).

Polymer **22** was processed in a similar manner to give conjugate **25** as white powder; ^1^H-NMR (D_2_O) δ: 5.64 (d, 1 H, *J*_1,2_ 4.7 Hz, H-1), 4.44 (d, 1 H, *J*_1',2'_ 7.7 Hz, H-2'), 4.42–4.36(m, 2 H, H-3, H-6a), 4.30–4.14 (m, 4 H, H-2, H-6a, OCH_2_OCO), 4.10–3.60 (m, 34 H, H-4, H-5, H-6a, H-6b, H-3', H-4', H-5', H-6a', H-6b', OCH, OCH_2_), 3.58 (dd, 1-H, *J*_2',3'_ 9.0 Hz, H-2'), 3.40–3.00 (m, 46 H, NCH_2_), 2.10–0.60 (m, 173 H, CH_2_, CH_3_). 

Polymer **23** was processed in a similar manner to give conjugate **26** as white powder; ^1^H-NMR (D_2_O) δ: 5.66 (d,1 H, *J*_1,2_ 4.3 Hz, H-1), 4.46 (d, 1 H, *J*_1',2'_ 7.8 Hz, H-1'), 4.44–4.16 (m, 2 H, H-3, H-6a), 4.28–4.18 (m, 4H, H-2, H-6b, CH_2_OCO), 4.06–4.00 (m,1 H, H-5), 4.38–3.62 (m, 31 H, H-4, H-3', H-4', H-5', H-6a', H-6b', OCH, OCH_2_), 3.56 (dd, 1 H, *J*_2',3'_ 9.8 Hz, H-2'), 3.40–3.00 (m, 40 H, NCH_2_), 2.10–0.80 (m, 140 H, CH_2_, CH_3_).

### 2.4. Enzymatic Galactosylation of HPMA-Lactose Polymers. Preparation of Trisaccharide Conjugates ***HPMA-n0***, ***HPMA-n1***, and ***HPMA-n2***

Polymer **24** (41.5 mg) was dissolved in water (506 μL) then HEPES buffer (43 μL) was added, followed by aq. DTT solution (0.4 M, 10.6 μL) and alkaline phosphatase (1 U/μL, 1.06 μL). UDP-Glc (12.6 mg, 0.02 mmol) was added and followed by crude fusion enzyme GalT/UDP-4'-epimerase [21] (32 μL) and the mixture was incubated at 37 °C overnight. Then it was treated with aq. 10% trichloroacetic acid (620 μL) and centrifuged. The solution was diluted with water and dialyzed against deionized water. The dialyzed solution was filtered through Milipore membrane (0.45 μm) and lyophilized to give the product as a white powder. ^1^H-NMR of the sample indicated the reaction was 70% complete. Enzymatic reaction was repeated to achieve complete conversion to give **HPMA-n0** as a white powder (32 mg); ^1^H-NMR (D_2_O) δ: 5.68 (bs, 1H, H-1), 4.96 (d, 1 H, *J*_1”,2''_ 3.5 Hz, H-1''), 4.52 (d, 1H, *J*_1,2_ 7.6 Hz, H-1), 4.46–4.34 (m, 3 H, H-3, H-5''), 4.28–4.18 (m, 2 H, H-2), 4.08–4.01 (m, 3 H, H-5, H-4', H-4''), 3.98–3.76 (m, 30 H, H-6a, H-6b, H-5', H-6a',H-6b', H-2'', H-3'', H-6a'', H-6b'', CHOH), 3.76-3.68 (m, 2H, H-4, H-3'), 3.60 (dd, 1 H, *J*_2',3'_ 9.7 Hz, H-2'), 3.60–3.00 (m, 50 H, NCH_2_), 2.1–0.6 (m, 198 H, CH_2_, CH_3_).

Polymer **25** was processed in a similar manner to give conjugate **HPMA-n1** as white powder; ^1^H-NMR (D_2_O) δ: 5.64 (bs, 1 H, H-1), 4.96 (d, 1 H, *J*_1'',2''_ 3.3 Hz, H-1''), 4.52 (d, 1 H, *J*_1,'2'_ 7.9 Hz, H-1'), 4.44 (m, 3 H, H-3, H-6a, H-5''), 4.30–4.16 (m, 4 H, H-2, H-6b, CH_2_OCO), 4.04 (bs, 3 H, H-5, H-4', H-4''), 4.00–3.62 (m, 32 H, H-4, H-3',H-5', H-6a', H-6b', H-2'', H-3'', H-6a'',H-6b'', CH,CH_2_), 3.60 (dd, 1-H, *J*_2',3'_ 8.7 Hz, H-2'), 3.40–3.00 (m, 43 H, NCH_2_), 2.10–0.80 (m, 157 H, CH_2_, CH_3_). 

Polymer **26** was processed in a similar manner to give conjugate **HPMA-n2** as white powder; ^1^H-NMR (D_2_O) δ: 5.66 (bs, 1H, H-1), 4.95 (d, 1 H, H-1''), 4.52 (d, 1 H, H-1'), 4.22–4.36 (m, 3 H, H-3, H-6a, H-5''), 4.28–4.16 (m, 4 H, H-2, H-6b, CH_2_OCO), 4.04 (bs, 3 H, H-5, H-4', H-4''), 3.98–3.56 (m, 38 H, H-4, H-2', H-3', H-5', H-6a', H-6b', H-2'', H-3'', H-6a'', H-6b'', CH, CH_2_), 3.40–3.00 (m, 49 H, NCH_2_), 2.10–0.80 (m, 175 H, CH_2_, CH_3_).

***N*-(2-(2-(2-azidoethoxy)ethoxy)ethyl)methacrylamide (27, AzMA).** To a solution of 2-(2-(2-azidoethoxy)ethoxy)ethanamine [16] (3.17 g, 18.2 mmol) in DCM (15 mL) and methacrylic anhydride (4.2 g, 1.5 eq) was added followed by Et_3_N (2 g, ~3 eq) and the mixture was stirred for 30 min. Chromatography on the silica gel in hexane—ethyl acetate (up to 70%) gave **27** (2.8 g, 63%). ^1^H-NMR (CDCl_3_) δ: 6.26 (s, 1 H, NH), 5.68–5.67 (m, 1 H, CH_2_=), 5.32–5.30 (m, 1 H, CH_2_=), 3.68–3.62 (m, 6 H, CH_2_), 3.59 (t, 2 H, *J* 4.7 Hz, CH_2_), 3.51 (dd, 2 H, *J* 5.5 Hz, *J* 10.8 Hz, CH_2_), 3.37 (t, 2 H *J* 4.8 Hz, CH_2_), 1.95 (m, 3 H, CH_3_); ^13^C-NMR (CDCl_3_) δ: 168.408 (C=O), 140.078 (C=CH_2_), 119.374 (C=CH_2_), 70.543 (CH_2_O), 70.236 (CH_2_O), 70.057 (CH_2_O), 69.843 (CH_2_O), 50.621 (C–N_3_), 39.333 (CH_2_–NH), 18.625 (CH_3_). ESI HRMS: *m*/*z*: calcd for C_10_H_18_N_4_O_3_Na ([M + Na]^+^): 265.1271; found 265.1271.

### 2.5. Typical RAFT Polymerization of Hydroxypropyl Methacrylamide Monomer

The statistical copolymerization **AzMA** and 2-hydroxylpropyl methacrylamide (HPMA) was performed at 70 °C in the presence of 4-cyanopentanoic acid dithiobenzoate (CTP) and 4,4'-azobis(4-cyanovaleric acid) (ACVA) as chain transfer agent and initiator, respectively. In a typical protocol, **AzMA** (0.18 mL, 0.4 mmol) and HPMA (1.1 g, 7.6 mmol) was dissolved in double distilled water containing a few drops of HCl (8 mL) followed by the addition of CTP (5 mg, 18 µmol) and ACVA (5 mg, 18 µmol) in 2-propanol (2mL) (targeted DP*_n_* 422, Targeted molecular weight 61 kDa). The mixture was purged with nitrogen for 45 minutes and flask was placed in oil bath for polymerization under nitrogen for 24 h. The polymerization was quenched using liquid nitrogen and polymer was precipitated in acetone. The molecular weight and molecular weight distributions of copolymers were determined using gel permeation chromatography in aqueous media.

### 2.6. General Procedure for Preparation of Polymeric Narrow Molecular Weight Heterobifunctional Ligands

An appropriate HPMA-AzMa copolymer (20–25 mg) and monomeric heterobifunctional ligand(**5**, **13** or **17**, 1.5–2.0 eq) were dissolved in water (1 mL) and neutralized with dry NaHCO_3_ (to pH: 8), then 1 M sodium ascorbate (25 μL) and 0.05 M (50 μL) were added and the solution was left on a tumbler for 2 days. The reaction was checked by IR for the presence of azide. When no azido group could be detected the reaction mixture was diluted with water and extensively dialyzed against deionized water with the addition of EDTA, then filtered and lyophilized. Product was obtained in the form of white powder.

**HPMA-16/5-A**; ^1^H-NMR (D_2_O) δ: 8.10 (s, 1 H, CH_triazol_), 7.80–7.50 (m, 24 H, NH_HPMA_), 5.62 (d, 1 H, *J*_1,2_ 4.8 Hz, H-1), 5.20 (bs, 2 H, OCH_2_), 4.94 (d, 1 H, *J*_1'',2''_ 3.9 Hz, H-1''), 4.60–4.53 (m, 2H, NCH_2_), 4.52 (d, 1 H, *J*_1',2'_ 7.7 Hz, H-1'), 4.42-4.35 (m, 3 H, H-3, H-6a, H-5''), 4.25–4.20 (m, 1 H, H-6b), 4.19–4.16 (m, 1 H, H-2), 4.05–4.00 (m, 3 H, H-5, H-4', H-4''), 3.96–3.56 (m, 51 H, H-4, H-2', H-3', H-5', H-6'a, H-2'', H-3'', H-6''a, H-6''b, 4 × OCH_2_, CHOH _HPMA_), 3.40–3.00 (m, 60 H, 2 × NCH_2_, NCH_2_ _HPMA_), 2.00–0.60 (m, 228 H, CH_3_, CH_2 HPMA_, CH_3 HPMA_).

**HPMA-44/5-A**; ^1^H-NMR (D_2_O) δ: 8.1 (s, 1 H, CH_triazol_), 5.62 (d, 1 H, *J*_1,2_ 4.9 Hz, H-1), 5.20 (bs, 2 H, OCH_2_), 4.94 (d, 1 H, *J*_1'',2''_ 3.9 Hz, H-1''), 4.60–4.54 (m, 2H, NCH_2_), 4.51 (d, 1 H, *J*_1',2'_ 7.7 Hz, H-1'), 4.42 (m, 3 H, H-3, H-6a, H-5''), 4.22 (m, 1 H, H-6b), 4.17 (m, 1 H, H-2), 4.06–3.99 (m, 3 H, H-5, H-4', H-4''), 3.97–3.86 (m, 20 H, H-6'a, H-3'',CHOH _HPMA_), 3.86–3.54 (m, 16 H, H-4, H-2', H-3', H-5', H-6'b, H-2'', H-6''a, H-6''b, 4 × OCH_2_), 3.70–3.00 (m, 38 H, 2 × NCH_2_, NCH_2_ _HPMA_), 2.00–0.60 (m, 136 H, CH_3_, CH_2_, CH_3 HPMA_).

**HPMA-35/10-A**; ^1^H-NMR (D_2_O) δ: 8.06 (s, 1 H, CH_triazol_), 5.62 (d, 1 H, *J*_1,2_ 4.9 Hz, H-1), 5.20 (bs, 2 H, OCH_2_), 4.94 (d, 1 H, *J*_1'',2''_ 4.0 Hz, H-1''), 4.60–4.53 (m, 2H, NCH_2_), 4.51 (d, 1 H, *J*_1',2'_ 7.7 Hz, H-1'), 4.42–4.34 (m, 3 H, H-3, H-6a, H-5''), 4.22–4.20 (m, 1 H, H-6b), 4.19–4.16 (m, 1 H, H-2), 4.04–4.00 (m, 3 H, H-5, H-4', H-4''), 3.96–3.86 (m, 13 H, H-6'a, H-3'',CHOH _HPMA_), 3.86–3.54 (m, 16 H, H-4, H-2', H-3', H-5', H-6'b, H-2'', H-6''a, H-6''b, 4 × OCH_2_), 3.90–2.90 (m, 28 H, 2 × NCH_2_, NCH_2_ _HPMA_), 2.00–0.60 (m, 100 H, CH_3_, CH_2_, CH_3 HPMA_).

**HPMA-20/15-A**; ^1^H-NMR (D_2_O) δ: 8.10 (s, 1 H, CH_triazol_), 5.62 (d, 1 H, *J*_1,2_ 4.6 Hz, H-1), 5.20 (bs, 2 H, OCH_2_), 4.95 (d, 1 H, *J*_1'',2''_ 3.9 Hz, H-1''), 4.60–4.53 (m, 2H, NCH_2_), 4.52 (d, 1 H, *J*_1',2'_ 7.7 Hz, H-1'), 4.42–4.36 (m, 3 H, H-3, H-6a, H-5''), 4.26–4.20 (m, 1 H, H-6b), 4.19–4.15 (m, 1 H, H-2), 4.05–4.01 (m, 3 H, H-5, H-4', H-4''), 3.96–3.86 (m, 12 H, H-6'a, H-3'',CHOH _HPMA_), 3.86–3.54 (m, 16 H, H-4, H-2', H-3', H-5', H-6'b, H-2'', H-6''a, H-6''b, 4 × OCH_2_), 3.90–3.00 (m, 26 H, 2 × NCH_2_, NCH_2_ _HPMA_), 2.20–0.60 (m, 90 H, CH_3_, CH_2 HPMA_, CH_3 HPMA_).

**HPMA-37/15-A**; ^1^H-NMR (D_2_O) δ: 8.10 (s, 1 H, CH_triazol_), 5.62 (d, 1 H, *J*_1,2_ 4.8 Hz, H-1), 5.20 (bs, 2 H, OCH_2_), 4.94 (d, 1 H, *J*_1'',2''_ 3.9 Hz, H-1''), 4.60–4.54 (m, 2 H, NCH_2_), 4.51 (d, 1 H, *J*_1',2'_ 7.7 Hz, H-1'), 4.42–4.34 (m, 3 H, H-3, H-6a, H-5''), 4.24–4.19 (m, 1 H, H-6b), 4.19–4.16 (m, 1 H, H-2), 4.04–4.01 (m, 3 H, H-5, H-4', H-4''), 3.96–3.86 (m, 11 H, H-6'a, H-3'',CHOH _HPMA_), 3.86–3.54 (m, 16 H, H-4, H-2', H-3', H-5', H-6'b, H-2'', H-6''a, H-6''b, 4 × OCH_2_), 3.90–3.00 (m, 22 H, 2 × NCH_2_, NCH_2_ _HPMA_), 2.20–0.60 (m, 80 H, CH_3_, CH_2 HPMA_, CH_3 HPMA_).

**HPMA-44/5-B**; ^1^H-NMR (D_2_O) δ: 8.10 (s, 1 H, CH_triazol_), 5.20 (bs, 2 H, OCH_2_), 4.94 (d, 1H, *J*_1'',2''_ 3.8 Hz, H-1''), 4.62–4.47 (m, 4 H, H-1, H-1', NCH_2_), 4.34 (dd, 1 H, *J*_5'',6''a _~ *J*_5'',6''b _~ 6.5 Hz, H-5''), 4.12–3.99 (m, 4 H, H-4', H-4'', H-4e_pyr_, H-6e_pyr_), 3.99–3.46 (m, 40 H, H-3, H-4, H-6a, H-6b, H-2', H-3', H-5', H-6'a, H-6'b, H-2'',H-3'', H-6''a, H-6''b, H-4a_pyr_, H-5_pyr_, H-6a_pyr_, OCH_2_, OCH _HPMA_), 3.35–2.96 (m, 45 H, H-2, NCH_2_ _HPMA_), 2.00–0.06 (m, 176 H, CH_3_, CH_2 HPMA_, CH_3 HPMA_).

**HPMA-35/10-B**; ^1^H-NMR (D_2_O) δ: 8.10 (s, 1 H, CH_triazol_), 5.20 (bs, 2 H, OCH_2_), 4.94 (d, 1H, *J*_1'',2''_ 3.8 Hz, H-1''), 4.63–4.47 (m, 4 H, H-1, H-1', NCH_2_), 4.34 (dd, 1 H, *J*_5'',6''a _~ *J*_5'',6''b _~ 6.4 Hz, H-5''), 4.12–4.00 (m, 4 H, H-4', H-4'', H-4e_pyr_, H-6e_pyr_), 3.98–3.46 (m, 41 H, H-3, H-4, H-6a, H-6b, H-2', H-3', H-5', H-6'a, H-6'b, H-2'',H-3'', H-6''a, H-6''b, H-4a_pyr_, H-5_pyr_, H-6a_pyr_, OCH_2_, OCH _HPMA_), 3.35–2.96 (m, 41 H, H-2, NCH_2_ _HPMA_), 2.00–0.06 (m, 160 H, CH_3_, CH_2 HPMA_, CH_3 HPMA_).

**HPMA-44/5-C**; ^1^H-NMR (D_2_O) δ: 8.10 (s, 1 H, CH_triazol_), 7.90–7.40 (m, 18 H, NH_ HPMA_), 4.94 (d, 1 H, *J*_1'',2''_ 3.9 Hz, H-1''), 4.59–4.48 (m, 3 H, H-1, H-1', OCH_2_), 4.34 (dd, 1 H, *J*_5'',6''a _~ *J*_5'',6''b _~ 6.5 Hz, H-5''), 4.23–4.16 (m, 2 H, CH_2_OCO), 4.14–4.06 (m, 2 H, H-4e_pyr_, H-6e_pyr_), 4.04–4.00 (m, 2H, H-4', H-4''), 3.98–3.49 (m, 54 H, H-3, H-4, H-5, H-6a, H-6b, H-2', H-3', H-5', H-6'a, H-6'b, H-2'', H-3'', H-6''a, H-6''b, H-4a_pyr_, H-5_pyr_, H-6a_pyr_, OCH, 6 × OCH_2_, OCH_HPMA_), 3.47–3.00 (m, 45 H, H-2, NCH_2_, NCH_2 HPMA_), 2.10–0.60 (m, 176 H, CH_3_, CH_2 HPMA_, CH_3 HPMA_).

**HPMA-35/10-C**; ^1^H-NMR (D_2_O) δ: 8.10 (s, 1 H, CH_triazol_), 7.90–7.40 (m, 13 H, NH_ HPMA_), 4.94 (d, 1 H, *J*_1'',2''_ 3.9 Hz, H-1''), 4.60–4.48 (m, 3 H, H-1, H-1', OCH_2_), 4.34 (dd, 1 H, *J*_5'',6''a _= *J*_5'',6''b_= 6.5 Hz, H-5''), 4.25–4.17 ( m, 2 H, CH_2_OCO), 4.14–4.06 (m, 2 H, H-4e_pyr_, H-6e_pyr_), 4.04–4.00 (m, 2 H, H-4', H-4''), 4.00–3.48 (m, 46 H, H-3, H-4, H-5, H-6a, H-6b, H-2', H-3', H-5', H-6'a, H-6'b, H-2'', H-3'', H-6''a, H-6''b, H-4a_pyr_, H-5_ pyr_, H-6a_pyr_, 6 × OCH_2_, OCH_HPMA_), 3.48–3.00 (m, 37 H, H-2, 2 × NCH_2_, NCH_2 HPMA_), 2.10–0.60 (m, 136 H, CH_3_, CH_2 HPMA_, CH_3 HPMA_).

### 2.7. Typical RAFT Polymerization of Acrylamide Monomers

The statistical copolymerization of 3-aminopropyl methacrylamide (APMA) and acrylamide (AA) was performed at 70 °C in the presence of 4-cyanopentanoic acid dithiobenzoate (CTP) and 4,4'-azobis(4-cyanovaleric acid) (ACVA) as chain transfer agent and initiator, respectively. In a typical protocol AA (2 g, 28 mmol) and APMA (0.25 g, 1.4 mmol) was dissolved in dimethylsulfoxide (DMSO) (10 mL) followed by the addition of CTP (8 mg, 29 µmol) and ACVA (2 mg, 7 µmol) (targeted DP*_n_* 977, Targeted molecular weight 70 kDa). The mixture was purged with nitrogen for 45 minutes and flask was placed in oil bath for polymerization under nitrogen for 24 h. The polymerization reaction mixture was quenched using liquid nitrogen and polymer was precipitated in acetone. The molecular weight and molecular weight distributions of copolymers were determined using aqueous gel permeation chromatography. 

**PAA-27/5.** Poly[acrylamide-co-(3-aminopropylmethacrylamide hydrochloride)] (M.W.: 27000; 5% of amine; 210 mg, 141 μmol) was dissolved in water (2.5 mL) and imidazole-1-sulfonyl azide [17] (118 mg, 563 μmol) was added followed by 0.4 M solution of CuSO_4_/H_2_O (12.5 μL) and pH of the solution was adjusted to 10 by addition of 5 M NaOH (200 μL). The reaction mixture was stirred at room temperature for 6 h, then dialyzed, filtered and lyophilized to provide the product as an off white powder (177 mg). IR: 2103.0 cm^−1^, ^1^H-NMR (D_2_O) δ: 3.44–3.35 (m, 2 H, NCH_2_), 3.32–3.12 (m, 2 H, NCH_2_), 2.38–1.98 (m, 16 H, CH_2 linker_, CH), 1.84–1.34 (m, 32 H, CH_2_), 1.2–1.08 (m, 3 H, CH_3_).

**PAA-72/5.** The title product was obtained from poly[acrylamide-co-(3-aminopropylmethacrylamide hydrochloride)] as described for preparation of **PAA-27/5** (M.W.: 72000; 5% of amine; 210 mg, 141 μmol) as described for **PAA-27/5**. The title product obtained as off white powder (156 mg). IR: 2103.9 cm^−1^. ^1^H-NMR (D_2_O) δ: 3.44–3.34 (m, 2 H, NCH_2_), 3.30–3.12 (m, 2 H, NCH_2_), 2.38–1.98 (m, 16 H, CH_2 linker_, CH), 1.82–1.34 (m, 34 H, CH_2_), 1.2–1.08 (m, 3 H, CH_3_).

**PAA-27/5-A.** Azide **PAA-27/5** (21.8 mg, 14.3 μmol) and **5** (23.5 mg, 28.3 μmol) were dissolved in water (1 mL) and pH adjusted to 8. Then 1 M sodium ascorbate (25 μL) and 0.05 M copper sulfate (50 μL). The reaction mixture was left on a tumbler for 2 days. Then it was dialyzed, filtered and freeze-dried to afford the polymer **PAA-27/5-A** as a white powder (35 mg). ^1^H-NMR (D_2_O) δ: 8.06 (s, 1 H, CH_triazol_), 5.62 (d, 1 H, *J*_1,2 _4.9 Hz, H-1), 5.18 (bs, 2 H, OCH_2_), 4.94 (d, 1 H, *J*_1'',2''_ 3.9 Hz, H-1''), 4.51 (d, 1 H, *J*_1',2'_ 7.8 Hz, H-1'), 4.50–4.35 (m, 5 H, *J*_6a,6b_ 12.0 Hz, H-3, H-6a, H-5'', NCH_2_), 4.23 (dd, 1 H, *J*_5,6b_ 5.1 Hz, H-6b), 4.18 (dd, 1 H, *J*_2,3_ 3.9 Hz, H-2), 4.04–4.01 (m, 3 H, H-5, H-4', H-4''), 3.94–3.89 (m, 2 H, H-6'a, H-3''), 3.85–3.60 (m, 16 H, H-4, H-2', H-3', H-5', H-6'b, H-2'', H-6''a, H-6''b, 4 × OCH_2_), 3.34–3.29 (m, 4 H, 2 × NCH_2_), 3.20–3.02 (m, 2 H, NCH_2_), 2.40–2.00 (m,16 H, CH_2 _, CH _PAA_), 1.80–1.30 (m, 31 H, CH_3_, CH_2 PAA_), 1.20–1.00 (m, 3 H, CH_3 MAA_).

**PAA-72/5-A.** Azide **PAA-72/5** (21.6 mg, 14.4 μmol) and **5** (24.3 mg, 29 μmol) were dissolved in water (1 mL) and reaction and work-up were performed as described for **PAA-27/5-A**. The tile **PAA-72/5-A** was obtained as a white powder (35.3 mg). ^1^H-NMR (D_2_O) δ: 8.10 (s, 1 H, CH_triazol_), 5.62 (d, 1 H, *J*_1,2_ 4.9 Hz, H-1), 5.18 (bs, 2 H, OCH_2_), 4.94 (d, 1 H, *J*_1'',2''_ 3.9 Hz, H-1''), 4.51 (d, 1 H, *J*_1',2'_ 7.7 Hz, H-1'), 4.49–4.34 (m, 5 H, *J*_6a,6b_ 11.8 Hz, H-3, H-6a, H-5'', NCH_2_), 4.23 (dd, 1 H, *J*_5,6b_ 5.1 Hz, H-6b), 4.18 (dd, 1 H, *J*_2,3_ 4.0 Hz, H-2), 4.05–4.00 (m, 3 H, H-5, H-4', H-4''), 3.94–3.89 (m, 2 H, H-6'a, H-3''), 3.85–3.60 (m, 16 H, H-4, H-2', H-3', H-5', H-6'b, H-2'', H-6''a, H-6''b, 4 × OCH_2_), 3.34–3.28 (m, 4 H, 2 × NCH_2_), 3.20–3.02 (m, 2 H, NCH_2_), 2.40–1.90 (m,19 H, CH_2_, CH _PAA_), 1.80–1.30 (m, 36 H, CH_3_, CH_2 PAA_), 1.20–1.00 (m, 3 H, CH_3 MAA_).

### 2.8. Biological Evaluation

The solid-phase binding-inhibition and Vero cell cytotoxicity-neutralization assays are fully described in previous reports [11,18,19]. In brief, C57BL/6-Tg(APSC)1Imeg transgenic mice [20], which exhibit liver-specific expression of human SAP at a stable circulating serum concentration of 30–40 μg/mL, were used in the Stx1-mediated Shigatoxemia experiments. All experiments were conducted in a double-blind, placebo-controlled manner. All animal protocols were reviewed and approved (Protocol Number MO4002) by the Faculty of Medicine, University of Calgary Animal Welfare Committee and were performed according to the Guidelines published by the Canadian Council on Animal Care, (Vol. 1, 2nd ed.). Human SAP-tg mice (*n* = 4–6 animals per group) were intravenously injected with a lethal dose(LD_100_ 20 ng/g of body weight) of Stx1 that was premixed in a total volume of 100 μL with a heterobifunctional inhibitor in physiological saline solution. Forty-eight h after challenge, the mice were monitored every 4 h and immediately euthanized by CO_2_asphyxia if signs of Shigatoxemia (lethargy) became apparent. On the 10th day, all surviving mice were euthanized.

## 3. Results and Discussion

### 3.1. Synthesis of Heterobivalent Polymers

**Scheme 1:**
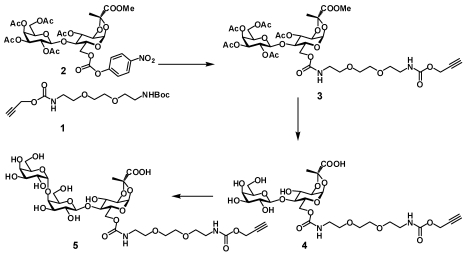
Synthesis of the Type A pendant ligand **5**.

Reaction of the *p*-nitrophenylcarbonate derivative of lactose **2** [11] with amine derived from Boc-protected linker **1** provided protected lactose intermediate **3** with a linker armed with a terminal alkyne residue. After deprotection and subsequent enzymatic glycosylation of **4** the heterobifuntional ligand **5** was obtained (Scheme 1).

**Scheme 2:**
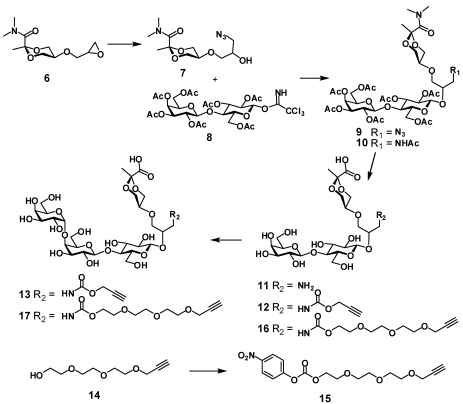
Synthesis of the Type B pendant ligands **13** and **17**.

The synthesis of two other heterobifunctional ligands **13** and **17** started with opening of the oxirane ring in cyclic pyruvate derivative **6** with sodium azide (Scheme 2). The resulting alcohol **7** was glycosylated with lactose donor **8**. Reduction of azide **9** followed by acetylation gave **10**, which was *N* and *O*-deacetylated with NaOH solution to give amine **11**. Acylation of the amino group in **11** with propargyl chloroformate gave carbamate derivative **12**, which was enzymatically α-galactosylated to give the desired ligand **13** ready for conjugation to a polymer. The enzymatic synthesis was previously described and employs a fusion protein that incorporates *gluco*-*galacto* epimerase and α-1,4-galactosyltransferase activities. This allows the use of the relatively cheap UDP-glucose donor to achieve the introduction of the terminal α-galactopyranosyl residue. Analogously, ligand **17** with a longer linker was obtained by first acylating **11** with activated carbonate **15** then enzymatically processing the disaccharide with attached tether **16** to give **17**.

**Scheme 3:**
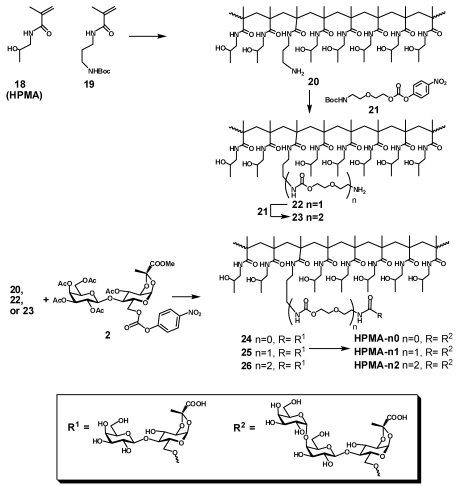
Synthesis of HPMA conjugates (Type A) by sequential extension of the linker-arm.

The first set of polymeric ligands was obtained by sequential elongation of the side chain linker in a single batch of co-polymer between *N*-(2-hydroxypropyl) methacrylamide (**18**, HPMA) and *N*-(3-*tert*-butoxycarbonylaminoxypropyl) methacrylamide (**19**) with cysteamine hydrochloride as a radical initiator. After deprotection of the product of polymerization, the HPMA polymer **20** randomly decorated with amino groups reacted with linker **21** to provide after deprotection polymer **22** with elongated side chains. The procedure was repeated to afford polymer **23** with twice elongated side chains (Scheme 3). The heterobifunctional pendant ligand was installed into the three homologous polymeric scaffolds **20**, **21** and **22** in two steps: first, amino groups in each polymer were coupled with the lactose pyruvate derivative **2** to provide after deacetylation the respective polymers **24**, **25** and **26**,each of which were enzymatically galactosylated to provide final products **HPMA-n0**, **HPMA-n1** and **HPMA-n2** correspondingly. 

**Scheme 4:**
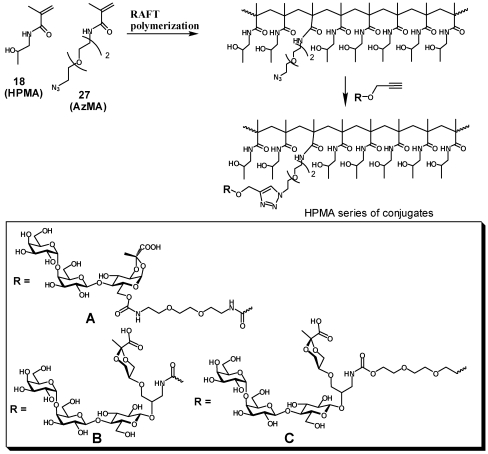
Synthesis of HPMA conjugates (Types A, B and C) with narrow molecular weight distribution by RAFT polymerization.

In order to assess influence of polymer size on the activity of heterobifunctional ligands the synthesis of polymeric scaffolds with randomly incorporated azide functionality and different molecular weight was performed by the reversible addition-fragmentation chain transfer process (RAFT). The RAFT copolymerization of HPMA with azide containing methacrylamide monomer (AzMA) was performed in aqueous conditions using 4,4'-azobis(4-cyanovaleric acid) as the initiator and 4-cyanopentanoic acid 4-dithiobenzoate as the chain transfer agent (Scheme 4, Table 1). Two polyacrylamide analogs **PAA-27/5PAA-72/5** with defined molecular weight were obtained by co-polymerization of acrylamide with 3-aminopropylmethacrylamide hydrochloride followed by azidination of amino group side chains using azidotransfer reagent, imidazole-1-sulfonyl azide [17]. 

The conjugation of the heterobifunctional ligands **5**, **13** and **17** to the azide-presenting scaffolds was accomplished via an efficient click reaction providing an array of polymeric heterobifunctional ligands: **HPMA-16/5-A**, **HPMA-44/5-A**, **HPMA-35/10-A**, **HPMA-20/15-A**, **HPMA-37/15-A**, **HPMA-44/5-B**, **HPMA-35/10-B**, **HPMA-44/5-C**, and **HPMA-35/10-C**, **PAA-27/5-A**, **PAA-72/5-A**.

**Table 1:** Polymeric scaffolds obtained by RAFT polymerization.

Polymer ^a^	Molecular Weight, Da	M_w_/M_n_	AzMA substitution, %
**HPMA-16/5**	15900	1.34	5
**HPMA-44/5**	43600	1.35	5
**HPMA-35/10**	35100	1.34	10
**HPMA-20/15**	19500	1.32	15
**HPMA-37/15**	36700	1.41	15
**PAA-27/5**	27000	1.29	5
**PAA-72/5**	72000	1.38	5

^a^ polymers are named according to the principle monomer; the indices indicate molecular weight in kDa and % substitution with azide monomer **AzMA**.

**Scheme 5:**
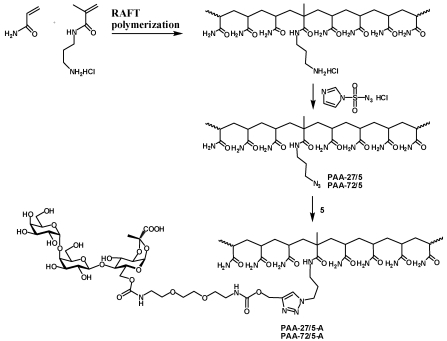
Synthesis of PAA conjugates (Type A) with narrow molecular weight distribution by RAFT polymerization.

### 3.2. Evaluation of Inhibitory Activity

Supramolecular inhibition is a novel strategy for the design of antagonists for pathogenic agents. The approach takes advantage of an endogenous protein such as an antibody or serum proteins of the innate immune system that serve as a template for presentation of a binding moiety specific to the target protein or cell [22,23,24,25,26]. In addition to providing a pre-organized scaffold and enhancing activity due to the multivalency effect the templating protein can function as an effector which initiates attack by the immune system or redirects the bound species away from susceptible organs. Thus, PolyBAIT-mediated interaction with SAP results in re-routing of Stx1 from kidney to the spleen and liver, where the toxin can be safely disposed [11]. 

In order to address the feasibility of using polymeric heterobifunctional inhibitors such as PolyBAIT as injectable drugs to treat patients infected with enterohemorrhagic *E. coli* two main issues have to be resolved, namely, replacing polyacrylamide with a more biocompatible polymer, and controlling the molecular weight of the polymer-carrier to be below the kidney excretion cut-off. As the polyacrylamide backbone replacement we chose *N*-(2-hydroxypropyl)methacryamide (HPMA). Since its original use as a blood plasma expander [27,28]. HPMA is the most extensively studied soluble polymer with a nondegradable backbone, which itself or as a copolymer have been employed in a broad variety of biomedical applications ranging from immunosuppressives, vaccines, cancerostatic drugs, imaging agents and targeting of gene delivery vectors [29]. 

**Figure 2:**
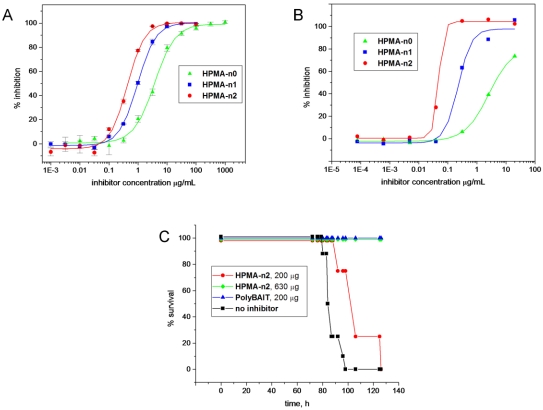
Biological evaluation of HPMA-based heterobifunctional inhibitors of Stx1. Panel **A**: inhibition of Stx1 binding to Gb3 analog-coated microtiter plates; Panel **B**: inhibition of intoxication of Vero cells with Stx1 (LD_100_ 25 ng/mL); Panel **C**: protection of from intoxication with Stx1 at LD100. Survival plot demonstrating the efficacy of **HPMA-n2** compared with the original PolyBAIT in preventing Stx1-mediated lethality in human SAP-transgenic mice. Mice received a single anterior dorsal injection of a lethal dose of Stx1 (LD_100_ 20 ng/g of body weight) that was premixed with a heterobifunctional inhibitor.

Preliminary evaluation of the feasibility of replacing acrylamide as the polymeric backbone in PolyBAITs was performed on a series of homologous polymeric inhibitors **HPMA-n0**, **HPMA-n1** and **HPMA-n2**. These differed only in the length of the spacer-arm and were identical in all other respects since all of them were prepared from the same batch of aminated polyHPMA by sequential elongation of the linker. Inhibition data for both solid phase assay and cell intoxication assay presented in Figure 2A and Figure 2B revealed a trend of increasing activity with elongation of the spacer. Additionally, direct correspondence between activities in both assays was in sharp contrast with other multivalent inhibitors, for which we previously observed significant reduction of activity upon transition from ELISA to cytotoxicity assays. At the same time, the inhibitory power of the highest activity conjugate **HPMA-n2** was still an order of magnitude lower than that of original polyacrylamide-based PolyBAIT (Table 2). 

Conjugate **HPMA-n2** was tested *in vivo* for its ability to prevent intoxication of transgenic mice, which express human SAP at levels similar to humans (~20–60 mg/L). At the concentration, which was sufficient for the original PolyBAIT preparation to completely protect mice from the effects of Stx1 (200 μg/mouse), administration of **HPMA-n2** resulted in 10–20 h delay of symptom onset, while complete protection was achieved at a 3 fold higher dose (Figure 2C). Again, lower inhibitory activity in the solid-phase and cytotoxicity assays directly translated into reduced efficacy *in vivo* (compare original PolyBAIT and **HPMA-n2**). This result confirmed the human SAP-dependant protective properties of tailored heterobifunctional polymers and the feasibility of transition of the methodology to a different polymer-carrier. Influences of length of the linker on the activity of the heterobifunctional polymer both in ELISA and cell culture assays suggest greater interference of the scaffold in HPMA polymers with formation of a supramolecular complex as compared with less bulky side chains in polyacrylamide. However, the question of how the nature of the polymer-carrier and its variable parameters such as molecular weight and payload influence the activity remained to be addressed.

Reversible addition–fragmentation chain transfer (RAFT) polymerization have previously been successfully employed to prepare HPMA copolymers with polydispersity of 1.1–1.8 (depending on molecular weight) containing methacrylamide monomers with a reactive group or cargo drug [30,31,32]. Such well defined copolymers with a narrow molecular weight range are the best candidates for *in vivo* and clinical testing of polymeric therapeutical and diagnostic agents. In the present work, we took advantage of the highly efficient “click reaction”, which enables conjugation of the active component to a polymer in aqueous media. The azido group of the **AzMA** monomer incorporated into HPMA backbone served as a reactive functionality, which permited nearly quantitative conjugation of carbohydrate ligands. 

Evaluation of a series of defined molecular weight heterobifunctional inhibitors was conducted in solid-phase ELISA assay (Table 2). Surprisingly, with the exception of polymer **HPMA-16/5-A** with the lowest molecular weight, all compounds of the HPMA series have shown very similar activities regardless of molecular weight and also regardless of the payload (percent incorporation) of the binding fragments. Furthermore, in the case of ligands containing spatially separated binding fragments in each pendant grouping (polymers of types B and C Scheme 4), elongation of the linker did not influence activity at all in sharp contrast with the fused analogs as discussed above. Noteworthy, in both HMPA and polyacrylamide series, increase of effective concentration of pendant heterobifunctional ligand either by increasing payload or size of the polymer was counterproductive: even if the effective dose remained the same the activity per pendant ligand reduced with increase of size and payload. The optimal activity could be achieved at relatively low payload and incorporation degree.

The crystal structure of Stx1 in complex with P^k^-trisaccharide [33] revealed three distinct P^k^-trisaccharide binding sites per B-subunit, of which site 2 has been shown to have the highest affinity [34]. Glucose moieties at the reducing end of bound P^k^-trisaccharides for site 1 and site 2 are located at approximately the same distance from the center pore of the B-pentamer and both are sufficient to span the distance between center pore of SAP and bound pyruvate ligand. However, when pyruvate is fused to glucose moiety (pendant ligand type A), only binding to site 2 provides the correct presentation of the pyruvate moiety to SAP. Site 3 is located too close the center pore and a heterobifunctional ligand would be unable to engage both proteins simultaneously at more than one or two points. Correct presentation of P^k^-trisaccharide as a part of Gb_3_ glycolipid has recently been shown to play an important role in the interaction of Stx1 with the cell surface [35]. Hence, only binding atsite 2 was considered in our design of pendant heterobifunctional ligands. A molecular model of the ternary complex mediated by a fused version of the heterobifunctional ligand (Figure 1) suggests that the exact alignment and orientation of each binding fragment to its respective protein should result in very close protein-to-protein contact leaving no space for a linker or polymeric scaffold. It is, therefore, unlikely that the scaffolding polymer and the linker occupy the space between Stx and SAP. Furthermore, when the trisaccharide is bound to Stx, the pyruvate moiety is now presented ~1.5–2 Å further toward the periphery and not exactly in register with the pyruvate-binding site on the SAP surface. Assembly of the ternary complex may require conformational distortions in the separate heterobifunctional ligands and entail entropic penalties. As a trade-off, the small gap between the multimeric receptors introduced by the short spacer in polymers of types B and C brings about easier passage of the scaffolding polymer between proteins and increases the number of possible microscopic complexes. Such additional freedom can add a favorable combinatorial entropy term to the binding free energy [36]. The optimum length of the spacer can probably be found for a particular supramolecular assembly.

**Table 2:** Activities of PolyBAITs.

Polymer	Pendant Ligand Type	Linker	Payload, %	IC_50_, μg/mL	IC_50_, μM ^a^
HPMA-n0 ^b^	Fused	Short	5	3.7	1.07
HPMA-n1	Fused	Long	5	0.98	0.27
HPMA-n2	Fused	Extra long	5	0.45	0.12
HPMA-16/5-A ^c^	Fused	Long	5	1.2	0.32
HPMA-44/5-A	Fused	Long	5	0.22	0.06
HPMA-35/10-A	Fused	Long	10	0.25	0.11
HPMA-20/15-A	Fused	Long	15	0.24	0.13
HPMA-37/15-A	Fused	Long	15	0.19	0.106
HPMA-44/5-B	Separate	Short	5	0.35	0.095
HPMA-35/10-B	Separate	Short	10	0.29	0.13
HPMA-44/5-C	Separate	Long	5	0.27	0.07
HPMA-35/10-C	Separate	Long	10	0.28	0.12
PAA-27/5-A	Fused	Long	5	0.019	0.008
PAA-72/5-A	Fused	Long	5	0.046	0.019

^a^ based on pendant ligand; ^b^ this and the following polymers are named according to the principle monomer followed by the number of extension linkers holding pendant ligand; ^c^ this and the following polymers are named according to the principle monomer; indices show molecular weight in kDa, % substitution with pendant ligands and type of conjugated unimeric ligand.

## 4. Conclusions

In summary, we have synthesized and evaluated a series of HPMA-based PolyBAITs, polymeric heterobifunctional ligands that inhibit Stx1 in a SAP-dependent manner. We observed that the inhibitory power of PolyBAITs in a solid-phase assay correlates with the cytotoxicity assay and efficacy *in vivo*. We demonstrate that the inhibition is moderately influenced by the structure of the pendant ligand and the length of the spacer but is practically independent of molecular weight and the polymer density of the glycans. Our results suggest that steric bulk of the side chains in the polymeric scaffold impedes assembly of a supramolecular complex with endogenous serum protein SAP and requires heterobifunctional ligands that allow for greater separation between specific binding fragments. 
